# Gastroparesis—An Often-Overlooked Sign of Multiple Sclerosis: Case Report

**DOI:** 10.1155/crii/1789381

**Published:** 2025-11-26

**Authors:** Breveenn Kukan, Kaylee Brown, Minh Chung, Steven Veselsky, Joshua Ferrell

**Affiliations:** Department of Family Medicine, HCA Memorial Health University Medical Center, Savannah, Georgia, USA

**Keywords:** autoimmune disease, case report, gastroparesis, multiple sclerosis, nystagmus

## Abstract

Multiple sclerosis (MS) is a chronic autoimmune disease and demyelinating disorder of the central nervous system (CNS) with diverse clinical presentations that can make the diagnosis challenging. In this case report, we describe a rare initial presentation of MS, mistaken for Type 1 diabetes mellitus (T1DM) impaired gastric motility. The patient is a 32-year-old female with a history significant for T1DM who presented with 3 days of intractable vertigo, nystagmus, and gait disturbance. She was discharged 2 days prior for intractable nausea and vomiting presumed to be due to impaired gastric motility called gastroparesis. There was no prior history of focal neurologic deficits. Her family history revealed extensive autoimmune diseases in multiple first-degree relatives. Physical examination suggested a peripheral lesion but could not rule out a central lesion. Magnetic resonance imaging (MRI) brain demonstrated white matter lesions in regions specific for MS. The patient experienced modest improvement with IV corticosteroids. Patients with T1DM have a threefold increase in the incidence of MS. While gastroparesis is an uncommon initial symptom of MS, this diagnosis should be considered, particularly when neurological deficits are present. This case underscores the importance of considering the enteric nervous system in patients with preexisting autoimmune conditions with new-onset neurological symptoms.

## 1. Introduction

Multiple sclerosis (MS) is a chronic autoimmune disease and demyelinating disorder of the central nervous system (CNS) that leads to severe physical, cognitive, and neurological impairments [1]. These symptoms often include visual disturbances, gait abnormalities, motor weakness, numbness, tingling, fatigue, emotional disturbances, learning difficulties, intestinal and urinary system dysfunction [2]. The disease pathogenesis involves inflammation in the CNS, specifically the destruction of oligodendrocytes and myelin sheaths, resulting in plaque formation within both white and gray matter [1]. The varied clinical presentations of MS require providers to have an attentive approach to diagnosis.

The etiology of MS remains incompletely understood, but it is thought to result from a combination of genetic and environmental factors. Genetic predisposition plays a role, as demonstrated by the ~25% risk of MS in monozygotic twins who share 100% genetic similarity [1]. The diagnostic process relies on medical history, neurological examination, and imaging techniques, such as magnetic resonance imaging (MRI), as well as cerebrospinal fluid (CSF) analysis through lumbar punctures and blood tests [1]. MS significantly reduces patients' quality of life and is associated with a shorter lifespan compared to the general population. However, early initiation of disease-modifying therapies improves prognosis and quality of life [3].

In this case report, we describe a rare initial presentation of MS, mistaken for Type 1 diabetes mellitus (T1DM) gastroparesis. Although uncommon, GI symptoms such as gastroparesis have been documented as a possible presenting feature of MS [4].

## 2. Case Presentation

The patient is a 32-year-old female with a past medical history significant for T1DM, Type 1 plasminogen activator inhibitor deficiency, and Pai syndrome. She was admitted to the hospital after 3 days of intractable vertigo, nystagmus, and gait disturbance. Notably, she had been hospitalized just 2 days prior for intractable nausea and vomiting, during which she was treated for euglycemic diabetic ketoacidosis (DKA) and underlying metabolic derangements. As part of her treatment for presumed gastroparesis, intravenous erythromycin was initiated and later transitioned to oral erythromycin upon discharge. However, the patient reported developing severe vertigo, described as “room spinning,” on the day of her discharge, and she was unable to fill her prescription for erythromycin due to access issues.

The vertigo persisted, worsening with head movements, and prompted her return to the emergency department. She described associated blurred vision but denied diplopia. Since the onset of symptoms, she experienced significant difficulty walking but denied dysarthria, dysphagia, aphasia, focal weakness, numbness, or tingling. There was no prior history of focal neurologic deficits such as vision loss or motor impairment. Additionally, she denied tinnitus, hearing loss, and urinary incontinence. Her family history revealed extensive autoimmune diseases in first-degree relatives, including lupus, Hashimoto's disease, and autoimmune hepatitis in her mother, and lupus in her sister. Her symptoms reportedly worsened with heat exposure known as the Uhthoff phenomenon.

Over the preceding year, the patient had sought emergency care seven times and had been hospitalized once for abdominal pain, nausea, and vomiting. These symptoms had been attributed to gastroparesis secondary to poorly controlled T1DM, although no formal diagnostic testing for gastroparesis had been completed. Plans had been made to consider a J-tube placement and gastric stimulator if her symptoms persisted. At home, she had been managing her nausea and vomiting with prochlorperazine and promethazine, though she reported only temporary relief. Erythromycin given during hospitalization did not improve nausea and vomiting.

**Figure 1:**
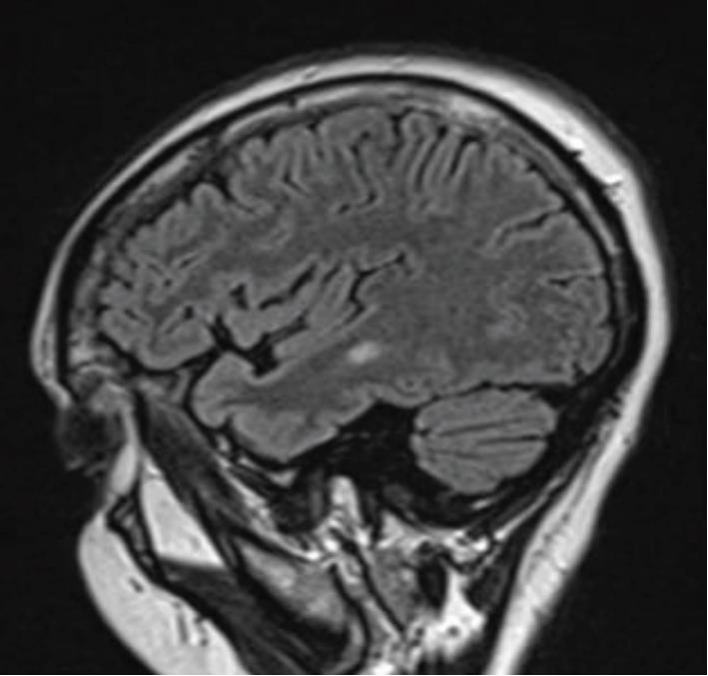
Sagittal T2 FLAIR MRI, series 9, image 5/48. Periventricular left temporal horn lesion.

**Figure 2:**
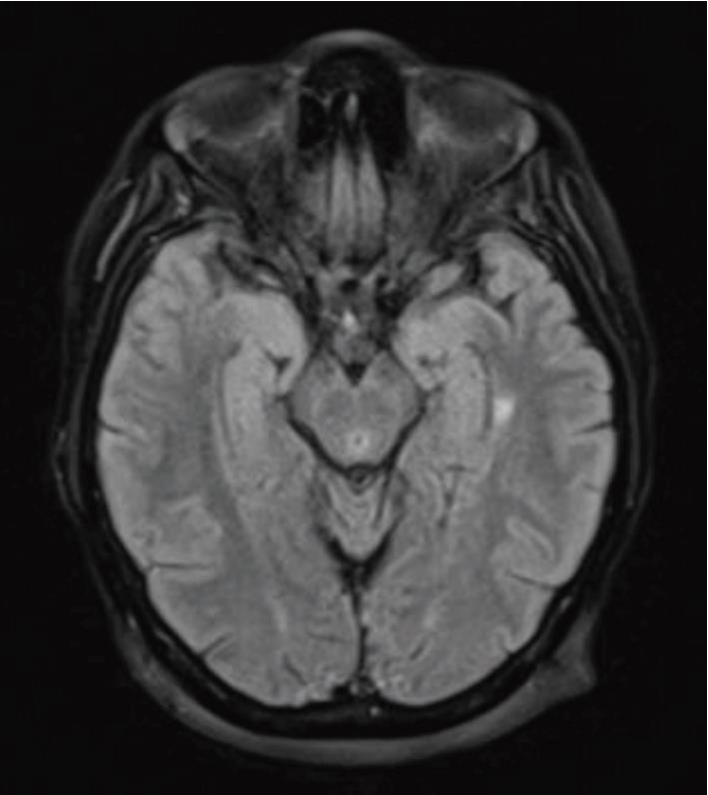
Axial T2 FLAIR MRI, series 7, image 13/32. Juxtacortical left parietal lobe lesion.

On admission, the patient was unable to ambulate due to gait imbalance. A HINTS exam revealed a positive head impulse test, bidirectional nystagmus, and a negative test of skew. While most findings suggested a peripheral lesion, the bidirectional nystagmus raised concern for a central process. Due to suspicion of an acute cerebrovascular event, the patient underwent an MRI brain without contrast, which demonstrated multiple fluid-attenuated inversion recovery (FLAIR) hyperintensities, suggestive of a demyelinating disease, involving the supratentorial, periventricular, and juxtacortical white matter without associated diffusion restriction. Subsequent MRI with and without contrast revealed two T2 hyperintense foci consistent with demyelinating disease adjacent to the left temporal horn and left parietal white matter, without enhancement (Figures 1 and 2).

Neurology was consulted, and the patient was started on intravenous methylprednisolone (250 mg daily for 3 days). Testing neuromyelitis optica (NMO) with AQP4 antibodies was negative. Physical therapy evaluation revealed decreased step length and cadence. A lumbar puncture was performed, and CSF analysis was positive for oligoclonal bands—further supporting a diagnosis of MS. The patient experienced modest improvement with IV corticosteroids and was discharged with instructions to follow up with outpatient neurology for continued management.

## 3. Discussion

MS cannot be ruled out as a cause of gastroparesis in patients with T1DM. In patients with T1DM, there is a threefold increase in the incidence of MS [5]. MS is an immune-mediated demyelinating disease that predominantly affects young adults, resulting in significant morbidity, including temporary and permanent disability, as well as a reduction in life expectancy by ~10–12 years [6]. While gastroparesis is an uncommon initial symptom of MS, this diagnosis should be considered, particularly in the absence of other apparent causes or when neurological deficits are present. This case underscores the importance of considering MS in patients with preexisting autoimmune conditions who develop new-onset neurological symptoms. Other less common initial presentations of MS include radiculopathy, headache, coma, seizure, isolated pain syndromes, diplopia, and changes in urinary habits [1].

The 2017 McDonald criteria for MS diagnosis were categorized based on the number of clinical attacks and the need for dissemination of lesions across space (DIS) and time (DIT) or the presence of oligoclonal CSF bands. Clinically isolated syndrome (CIS) can be diagnosed with one clinical attack and dissemination in space if one or more lesions is evident on MRI in two or more areas of CNS: periventricular, cortical, juxtacortical, infratentorial, or spinal cord. Dissemination in time can be substituted with the presence of oligoclonal bands in CSF to diagnose MS [7]. In 85% of MS cases, the patient starts off with CIS indicative of emerging CNS demyelination.

The 2017 McDonald's criteria for MS were used, specifically the CIS criteria, to diagnose MS in this patient. The patient showed evidence of early emerging MS consistent with CIS. She had a single clinical attack and dissemination in space with one or more lesions in two or more areas of the CNS (one in the periventricular region and one in the juxtacortical region). Since this was her first apparent attack due to no previous MRI done before this hospitalization, the dissemination in time was substituted with the presence of oligoclonal bands in CSF performed via lumbar puncture that is considered an acceptable substitute according to the 2017 McDonald's criteria to diagnose MS.

The lack of enhancement of the hyperintense lesions on MRI likely reflects the early stages of inflammatory demyelination, where disruption of the blood–brain barrier has not fully developed, and the acute enhancement seen with vasogenic edema is absent [8]. Dissemination in space was demonstrated by the presence of lesions in two of the four characteristic regions for MS: the periventricular and juxtacortical regions [9]. The anterior temporal horn lesion was found to be highly significant for MS when compared to non-MS conditions [10].

This case also highlights the challenges in distinguishing MS-related GI symptoms from those caused by T1DM. If the patient's gastroparesis were solely due to T1DM, erythromycin treatment would likely have resulted in more substantial improvement, which it did not. Over the past 2 years, the patient's HgA1c was improving with HgA1c 2 years ago at >15.5%, 9 months ago at 12.6%, and 5 months ago at 11.9%. The patient's kidney function was also improving with microalbumin/creatinine ratio going from 38 mg/g 2 years ago to within normal limits 5 months ago indicating improved kidney organ function. If the gastroparesis was due to T1DM, the patient would have experienced symptomatic GI organ relief of abdominal pain, nausea, and vomiting secondary to improved diabetes control instead of a worsening clinical picture. The underlying error being the misattribution of gastroparesis to T1DM instead of MS, given the context of new-onset neurological symptoms during the admission.

Emerging research suggests that the enteric nervous system may be involved in MS pathogenesis. There is precedent in that a mouse study of MS demonstrated neuronal degeneration in the GI tract leading to reduced GI motility before the overt onset of CNS lesions and neurological deficits as seen in this patient [11].

Prompt recognition of MS is crucial to initiate early treatment, which can prevent permanent disability and mitigate harm from potential misdiagnosis. Consistent with findings from a systematic review on gait abnormalities in MS, this patient exhibited decreased stride length and walking speed [12]. This case underscores the importance of maintaining a high index of suspicion for MS in patients with autoimmune conditions presenting with atypical or unexplained neurological and systemic symptoms. Early diagnosis and intervention can significantly improve patient outcomes and quality of life.

## 4. Conclusion

MS is a significant cause of morbidity and years of potential life lost, particularly among younger populations. Its diverse and variable presenting symptoms can make initial diagnosis challenging. In this case, the patient's comorbidities and atypical presentation led to a delay in diagnosis until characteristic lesions were incidentally identified on MRI. The gastro-motility disorder, manifesting as intractable nausea and vomiting, was initially misattributed to T1DM rather than the less commonly recognized association with MS.

This case highlights that reduced gastric motility can be an early, underappreciated symptom of MS and may serve as a precursor to overt neurological symptoms. A thorough family history and a detailed timeline of neurological and systemic symptoms are crucial for recognizing the connection between seemingly unrelated effects. In the minds of clinicians, the prominence of the enteric nervous system must be elevated to the level of importance of the CNS to allow seamless integration of nervous system findings. Early identification of MS through clinical and radiological findings allowed for the prompt initiation of first-line steroid therapy, which was effective in managing the patient's neuroimmunological symptoms and improving her overall condition.

This case emphasizes the importance of maintaining a high index of suspicion for MS in patients with autoimmune comorbidities and atypical presentations, ensuring timely diagnosis and treatment to improve patient outcomes.

## Data Availability

Data sharing is not applicable to this article, as no datasets were generated or analyzed during the current study.
